# Editorial

**Published:** 1853-09

**Authors:** 


### THE MEDICAL EXAMINER.

### PHILADELPHIA, SEPTEMBER, 1853.

### YELLOW FEVER IN PHILADELPHIA.

Since the August number of the Examiner went to press, our community has been startled by an unexpected visit from the yellow fever, which for upwards of thirty years has been an entire stranger to our city, so proverbial for its cleanliness and health.

Fortunately for Philadelphia, this dread enemy no sooner showed its malignant foot-prints on our borders, than the Board of Health, in the exercise of their prerogative, and with a commendable energy, instituted a most rigid investigation in the neighborhood where the first case appeared, and with the sanitary besom swept away, it is to be hoped, whatever nuisances were fruitful in concealing and propagating that poisonous *materiel*, be it malaria, unhealthy gases, animalcula, fungi, or what not, which are ever so productive of epidemics.

To this prompt and energetic action, coupled with the efficient municipal arrangements for preserving the cleanliness of the city, in its streets, gutters, and sewers, together with the abundant supply of water, must be attributed in a great measure the present arrest of the disease, and hence the preservation of the health of its 450,000 inhabitants, from the inroads of a fearful pestilence.

Thus far the fever has been confined to a very limited district, embracing an area of not more than 600 yards in length by 200 in breadth, bounded by Union street on the north, Second street on the west, Almond street on the south, and the Delaware front on the east, a neighborhood by no means densely peopled; but in many places the houses are confined and filthy, the Delaware avenue defective in surface drainage, the cellars in the vicinity damp and subject to an overflow during the flood and ebb of the tide waters of the Delaware, rendering the neighborhood insalubrious from excessive moisture, and the exhalation of noxious vapors, the product of animal and vegetable decomposition.

The origin of this fever may be involved in some doubt and obscurity. Whether from a specific germ imported in the barque Mandarin, or the product of her putrid bilge water, or of spontaneous birth in that particular locality where the irruption first manifested itself, still remains a perplexing question to solve. One circumstance connected with its history is clear, viz: that no yellow or malignant fever, or any epidemic, pervaded our city prior to the arrival of the barque Mandarin. This unfortunate vessel arrived here, all well, from Cienfuegos, Cuba, after a passage of seventeen days, on the 13th of July last, and hauled to at a wharf between Lombard and South streets. Six days after, the first outbreak of fever of a malignant type showed itself in that immediate vicinity. From this point it spread itself in about one month over the district above named.

It may be proper here to state that the Mandarin came from a port in the West Indies, where "a few cases of small pox and fever" prevailed; that she lost two of her crew from "fever" on the passage; that her bilge water was in a very foul condition, and notwithstanding she was detained at the Lazaretto, for cleansing purposes, yet, after opening her hatches and discharging her cargo, consisting of sugar and molasses, the bilge water was exceedingly offensive, and before she was removed by the Board of Health, the stench became intolerable.

During the two weeks that this vessel remained in the vicinity of South street wharf, the mean of the thermometer was 79½°.

As already hinted, the first case of suspicious fever happened on the 19th of July, six days after the arrival of the Mandarin, in the person of Joseph Sharp, a young and healthy carman, at South street ferry. On the next day, the 20th, five cases were reported, on the 21st four cases, on the 22d one case, on the 23d two, on the 25th three, on the 26th one, on the 27th one, on the 29th three, on the 30th two, on the 6th of August one, on the 9th two, on the 10th one, and so on up to the 20th—all of them, with the exception of the 21st, being clearly and distinctly traced as having their origin either by a residence in or a direct communication with the infected district.

The following is a list of the cases, in the order of their occurrence, up to the 20th of August:

| | Date of attack. | Died. |
|---|---|---|
| 1. Joseph Sharp | July 19th | July 26th. |
| 2. Geo. Robinson | " 20th | " 23d. |
| 3. Thos. D. Koehler | " " | " 27th. |
| 4. G. W. Kerkeslager | " " | " 25th. |
| 5. Mrs. Kerkeslager | " " | " 26th. |
| 6. Charles Burrows | " " | " 22d. |

| | | Date of attack. | Died. | |
|---|---|---|---|---|
| 7. | Fred. H. Kellog . | July 21st . | July 23d. | |
| 8. | Fanny Martin . . | " 21st . | " 26th. | |
| 9. | J. M. G. Koehler . | " " . | | Recovered. |
| 10. | Paulina Koehler . | " " . | | " |
| 11. | Honora Stanton . | " 22d . | July 27th. | |
| 12. | Silas Green . . | " 23d . | | Recovered. |
| 13. | R. N. Campbell . | " " . | | " |
| 14. | John Shellcott . | " 25th . | July 30th. | |
| 15. | Rosanna McKean . | " " . | " " | |
| 16. | John White . | " " . | | Recovered. |
| 17. | David Murray . | " 26th . | | " |
| 18. | James Markley . | " 27th . | | " |
| 19. | James Clements . | " 29th . | Aug. 1st. | |
| 20. | Mrs. Mullen . . | " " . | " 5th. | |
| 21. | John Haslett . . | " 29 . | " 2d. | |
| 22. | Mary Bulger . . | " 30th . | " 3d. | |
| 23. | Ann Jane Stewart . | " " . | " 13th. | |
| 24. | Mary Williams . | Aug. 6th . | " 12th. | |
| 25. | John S. Williams . | " 9th . | " " | |
| 26. | Betsey Stephens . | " " . | " 13th. | |
| 27. | Ellen Boland . . | " 10th . | " 15th. | |
| 28. | James Riley . . | " 13th . | " 15th. | |
| 29. | Bridget Tibley . | " 14th . | " 22d. | |
| 30. | Oliver Pitcher . | " 15th . | " 18th. | |
| 31. | James Genton . | " 15th . | " 27th. | |
| 32. | Mary Carrigan . | " 17th . | " 19th. | |
| 33. | " " (daughter) | " " . | " 20th. | |
| 34. | Mary Turner . | " 18th . | " " | |
| 35. | John Brennock . | " " . | " 19th. | |
| 36. | John Carrigan . | " 20th . | " 22d. | |
| 37. | Catharine Handson . | " . | " 22d. | |
| 38. | Edward Carrigan | | | |

Of the above 38 cases, 31 of them died, 24 of which were accompanied with black vomit.

The 21st case, of John Haslett, the drayman, cannot be traced to any portion of the infected district, although he stood on Chesnut street wharf, and was engaged daily in hauling goods along the eastern front of the city, and may perchance have been within the range of infection.

In enumerating all the facts in connexion with the appearance of this fever, it must not be overlooked, that at the dock fronting South street there is the open mouth of one of the sewers of the city, belching forth at all times its decomposing compounds of filth, which, when the tide is low, being exposed, must contaminate the immediate atmosphere with foul and unwholesome exhalations.

The question may be agitated by some one who has not witnessed a single case of this disease, "Is it yellow fever?" In answer, we would reply, that the most reliable evidence has been furnished of its being genuine yellow fever. Prof. Samuel Jackson saw two of Dr. Gegan's cases, and had one of his own; in all of them he recognized the same enemy that visited our city in 1820. Other gentlemen, who had seen the disease in the South, and in the West Indies, at once regarded the fever to be similar in all its features.

It was ushered in by intense headache, pains in the loins and extremities, distress at the epigastrium, nausea and vomiting, tongue soft and white, with red edges, eyes of a pink or yellow color, skin assuming a yellow tinge, in some instances petechiæ; the fever lasting for three and sometimes four days, and then remitting; the patient feeling better, and in some cases says he is well; but in a few hours, prostration comes on rapidly, with vomiting of black matter, in which, under the microscope, blood corpuscles have been discovered; the ejections from the bowels are thin, watery and dark in their appearance, with hemorrhages. In the fatal cases, no reaction of the system comes on, and the patient dies on the third, fourth or fifth day, and almost invariably the whole surface of the body becomes of a deep jaundice color.

A post mortem, where it has been made, reveals the usual appearances found after death, in those who have died of yellow fever, viz.—softening of the mucous membrane of stomach with patches of inflammation, the liver contracted and invariably of a light yellow or box wood color. In several instances the peculiar black fluid, called black vomit, was found in the stomach or intestines.

Where reaction follows the remission of the fever, the recovery has generally been rapid. In Dr. Jackson's case, the 22d, there was no steady reaction after the third day of attack, and no black vomit, while the patient lingered until the fourteenth day and then died. The yellow skin and pink eye, with oppression and great distress over the epigastrium and heart, were prominent symptoms in this case.

Besides the cases above numerated, there have been several deaths from fever, in the immediate vicinity of the infected district, which bore some slight resemblance to those cases classed under the head of Yellow Fever; but as the disease has not as yet assumed an epidemic form, it is presumed they were no more than ordinary instances of bilious fever, which usually makes its appearance about this period of the year.

We have endeavored to obtain a list of all the well authenticated cases which have occurred, and we believe we have succeeded in securing the most reliable information on this interesting topic. If there have been other cases, they were kept from the public, and therefore we had no access to them.

Since the above was in type, a few more cases have occurred, all of which, with a few exceptions, may be traced to that section of the city where the disease first made its appearance.

We may congratulate ourselves that no epidemic is at this time prevalent in our city The cases of fever that are to be found, are few in number, and are beginning to assume the ordinary bilious character of our Autumnal diseases.

Indeed our city is healthy; comparing the weekly statement of deaths with those of last year, they will be found rather under, than above the mortality of the same season in 1852.

[We are indebted for the above very interesting account of the recent cases of yellow fever in this city, to our esteemed correspondent, Dr. WILSON JEWELL. A more detailed notice of the earlier of these cases was read by Dr. Jewell before the August meeting of the College of Physicians, and will appear in the Transactions of that body.—EDS. EXAMINER.]

---

It will be seen, from the list of Books received, prefixed to the present number, that an unusual amount of new publications has been, within a short period, placed upon our table. Our limits prevent an immediate notice of all, but we hope to be able to do them justice in the October number.

